# Lessons learned from metabolomics in cystic fibrosis

**DOI:** 10.1186/s40348-015-0020-8

**Published:** 2015-10-20

**Authors:** Marianne S. Muhlebach, Wei Sha

**Affiliations:** Department Pediatrics, Division Pulmonology, UNC Chapel Hill 430 MacNider, CB 7217, Chapel Hill, NC 27599-7217 USA; Bioinformatics Services Division, University of North Carolina at Charlotte, Kannapolis, NC 28081 USA

**Keywords:** Cystic fibrosis, Metabolomics, Bronchoalveolar lavage, Exhaled breath, Sputum, Mass spectrometry

## Abstract

Cystic fibrosis is a mono-genetic multi-system disease; however, respiratory manifestations cause the main morbidity and mortality where chronic bacterial infections lead to bronchiectasis and ultimately respiratory failure. Metabolomics allows a relatively complete snapshot of metabolic processes in a sample using different mass spectrometry methods. Sample types used for discovery of biomarkers or pathomechanisms in cystic fibrosis (CF) have included blood, respiratory secretions, and exhaled breath to date. Metabolomics has shown distinction of CF vs. non-CF for matrices of blood, exhaled breath, and respiratory epithelial cultures, each showing different pathways. Severity of lung disease has been addressed by studies in bronchoalveolar lavage and exhaled breath condensate showing separation by metabolites that the authors of each study related to inflammation; e.g., ethanol, acetone, purines. Lipidomics has been applied to blood and sputum samples showing associations with lung function and *Pseudomonas aeruginosa* infection status. Finally, studies of bacteria grown *in vitro* showed differences of bacterial metabolites to be associated with clinical parameters. Metabolomics, in the sense of global metabolomic profiling, is a powerful technique that has allowed discovery of pathways that had not previously been implicated in CF. These may include purines, mitochondrial pathways, and different aspects of glucose metabolism besides the known differences in lipid metabolism in CF. However, targeted studies to validate such potential metabolites and pathways of interest are necessary. Studies evaluating metabolites of bacterial origin are in their early stages. Thus further well-designed studies could be envisioned.

## Introduction

Cystic fibrosis (CF) or mucoviscidosis is a multi-system monogenetic disease caused by mutation of the cystic fibrosis transmembrane regulator (CFTR) gene leading to abnormal folding and function of the CFTR protein, which is a chloride/bicarbonate channel. Absence of a functional protein leads to dehydration and acidification of glandular and other secretions. Lung manifestations are abnormal mucus viscosity and rheology, abnormal mucociliary clearance with propensity to bacterial infection, and increased inflammation [1]. Gastrointestinal manifestations of CF include pancreatic insufficiency, malabsorption, and abnormal intestinal motility often associated with bacterial intestinal overgrowth. Intestinal obstruction in the newborn period and distal intestinal obstruction syndrome (DIOS) in older patients can occur secondary to abnormal viscosity and probably also due to intestinal mucus obstruction. Metabolic abnormalities that are not considered to be a consequence of malabsorption have been reported for essential fatty acids, for instance docosahexaenoic acid and linoleic acid [2], and cholesterol and triglyceride pathways [3].

As early detection and initiation of therapies result in better outcomes, diagnosis occurs by newborn screening in many countries. Definitive diagnosis is made by sweat test, showing elevated chloride concentration in CF. Metabolomic technology in CF has been used for biomarker detection and identification of potential pathomechanistic changes to advance novel therapeutic approaches.

Metabolomics or metabolomic profiling is the comprehensive assessment of endogenous and exogenous metabolites in small volumes of bodily secretions and tissue samples. The detectable metabolites include lipids, carbohydrates, peptides, and proteins with different molecular size and charge of either endogenous or exogenous origin. Untargeted metabolomic profiling has been used for assessment of pathways in the context of disease diagnosis, insight into normal or pathologic processes, comparison of groups and disease processes, and normal events such as aging. The most widely used and sensitive technical approaches for metabolomics include nuclear magnetic resonance (NMR), which can be used without sample pre-separation, and mass spectrometry (MS) methods coupled to different extraction methods. Extraction methods used for sample preparation include gas or liquid chromatography (GC or LC) and capillary electrophoresis (CE). While extraction methods for MS may potentially bias the detection of metabolites, MS has the advantage of being more sensitive compared to NMR techniques. Another technique that is currently considered the state-of-the-art instrument secondary to its wider dynamic range is ultra performance liquid chromatography-mass spectrometry (elevated energy) (UPLC-MS(E)) or UPLC coupled to MS via electrospray ionization (ESI) [4]. This method provides higher selectivity and sensitivity compared to other methods.

This review will focus on untargeted metabolomic profiling for biomarker or pathway discovery although many further studies, not referenced here, have used sensitive NMR or MS technologies for targeted metabolomics, i.e., measuring known metabolites.

## Review

### Metabolomic studies in CF

**Fig. 1:**
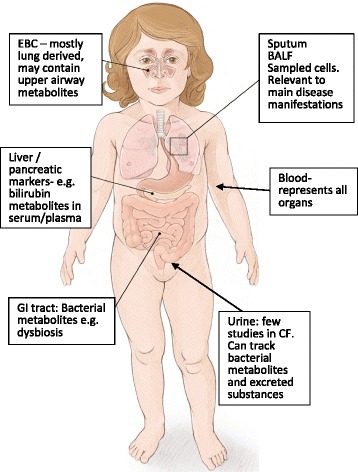
Sample types reflective of specific or general disease manifestation

Patient-based studies have utilized blood, sputum, bronchoalveolar lavage fluid (BALF), exhaled breath condensate (EBC), and urine as biological samples to evaluate differences between CF and non-CF subjects, to evaluate markers of disease severity or to gain novel insight into pathophysiology. Mechanistic studies used cell cultures and bacteria derived from CF lung infection as substrates. Figure 1 provides a schematic.

#### Blood-based studies

Blood samples can be obtained relatively noninvasively from subjects at all ages; however, the measured metabolites may be from any organ and may lack sensitivity for the compartmentalized CF lung disease. Serum or plasma should not be used interchangeably given significant differences that have been described in a systematic analysis in healthy, fasting subjects, where serum was found being less sensitive to incubation procedures and revealing a higher number of metabolites compared to the concomitantly obtained plasma [5]. Comparison of serum metabolomic profiles from 31 children with CF to age- and gender-matched children with other respiratory diseases showed differences in several metabolites and pathways linked to mitochondrial function including decreases in ketone bodies and medium chain carnitines. Further, several branched-chain amino acids were decreased in CF compared to non-CF suggestive of muscle wasting. Differences in bile acids and presumably bacterial-derived metabolites from the tryptophan pathway could be related to intestinal dysbiosis [6]. Laguna et al. evaluated plasma metabolomics in 25 subjects with CF comparing patient samples collected during exacerbation vs. clinical stability. Of the 398 identified metabolites, five metabolites were significantly lower during exacerbation compared to clinical stability. The affected pathways were related to nucleotide metabolism (hypoxanthine and N4-acetylcytidine), amino acid metabolism (N-acetylmethionine), carbohydrate (mannose), and steroid metabolism (cortisol). Principal component analysis using these five metabolites provided 79 % separation [7]. Given the known differences in lipid content of cells and fluids in CF, e.g., n-6 and n-3 fatty acids [8] and cholesterol [9], two studies used lipidomics for specific assessment of phospho- and lysolipids following organic extraction of plasma. Eighteen healthy children, 33 children with mild lung disease, and ten children with severe lung disease of CF were included [10]. Eleven peaks contributed to separation between CF and non-CF and four peaks were differentially displayed in mild vs. severe CF. A follow-up study evaluated potential correlations of plasma lipidomics with severity of lung disease and chronic *Pseudomonas aeruginosa* infection status. Plasma was obtained from 44 F508del homozygote patients at two time points, 3 years apart, to track disease progression. Twelve free fatty acids correlated with FEV_1_ and six lipids showed associations with *P. aeruginosa* infection [11].

#### Sputum-based studies

Lipidomics has also been applied to sputum obtained from 16 adult patients, the majority of whom were *P. aeruginosa* infected. Using three different extraction methods, a range of lipid mediators was detected including those related to cyclooxygenase and lipoxygenase pathways [12]. Some of these, e.g., an epoxide of linoleic acid and LTB4, showed correlations to lung function either individually or in multivariate partial least squares analyses. Notably, the high bacterial load and various proteases in CF sputum, extraction methods, and storage could affect metabolites. Recently, Zhao et al. showed differences in LC-MS metabolomic signatures after storage at 4° but not at −20° and −80° for at least 28 days [10, 13].

#### Bronchoalveolar lavage

Bronchoalveolar lavage (BAL) is performed in many institutions in children who cannot expectorate. Inflammation normalized to infection is already elevated in young children with CF compared to non-CF disease controls [14] and neutrophil elastase in BAL fluid (BALF) may be an early biomarker [15]. Using ^1^H NMR, Wolak et al. evaluated BALF from 11 subjects with CF undergoing clinically indicated procedures. A larger number of metabolites were detectable in samples with higher levels of inflammation as determined by relative and absolute neutrophil count. The selected and identified peaks were derived predominantly from amino acids, lactate, and acetone. The authors acknowledged the challenge of dilution factor in BALF and used different methods to account for this, e.g., normalization to acetone and statistical processing. Quantitative fitting of alanine, taurine, valine, and lactate resulted in clear separation between low and high inflammation samples [16].

Subsequent evaluations compared BALF from CF to non-CF disease controls using UPLC-QtofMS [17]. The discovery set of 25 samples revealed 338 peaks that were associated with inflammation defined by neutrophil count. Named metabolites included those from purine, polyamine, and nicotinamide pathways and metabolites related to protein degradation. Thirty markers were validated using targeted MS in a new set of BALF samples derived from pediatric subjects with CF, healthy non-smoking or smoking adults, and subjects with COPD. Metabolites related to purine metabolism and protein degradation were correlated to neutrophilic inflammation in the various sample types and diseases, and several markers correlated with lung function [17].

#### Exhaled breath—breathomics

A less invasive method than BAL is measurement of metabolites in exhaled breath collected as condensate of water-soluble markers (EBCs) present in epithelial lining fluid and/or as volatile organic compounds (VOCs). Challenges include variability of EBC production between individuals, the lack of a marker of dilution, and potential influence of different collection devices [18]. Only few data are reported on metabolomic profiling of EBC compared to measurement of specific markers using either mass spectrometry or NMR methods. In a cross-sectional study, Montuschi et al. evaluated if the NMR spectroscopy profiles followed by identification of selected compounds in EBC differentiated stable (*n* = 29) from non-stable (*n* = 24) patients with CF [19]. Among the 11 most differentiating metabolites using PLS-DA, four (acetate, ethanol, 2-propanol, and acetone) separated the CF from healthy group. Seven metabolites were most relevant for distinguishing stable vs. exacerbation samples in CF, again this could be reduced to four metabolites (ethanol, acetate, 2-propanol, and methanol) as a panel without significant loss in sensitivity and specificity. Importantly, the study provided careful control measures including within day and between-day repeatability in disease and healthy subjects and external validation in a set of patients from a different CF center. The same group of authors more recently reported discriminatory NMR spectra of EBC in CF compared to primary ciliary dyskinesia (PCD) [20]. Both the discovery and the validation group of CF and control subjects had been included in the prior analyses, but repeat measures confirmed identification of the same metabolites. Seven metabolites, including ethanol and acetate, discriminated CF from PCD. When only three metabolites—acetoin, lactate, and methanol—were included in the panel, there was only a 6 % reduction in *R*^2^. Short-chain fatty acids, ethanol, and methanol discriminated both diseases compared to healthy subjects. The authors speculated that several of these markers may be related to inflammation.

VOCs are produced during most metabolic reactions and markers present in exhaled breath are either derived from the lung itself or metabolites diffusing through the epithelium. Analyses can be performed using GC-MS or, to make it more affordable and portable, metallic sensors that detect pattern of volatiles [21]. Robroeks et al. compared VOC in breath obtained from 57 healthy controls and 48 subjects with CF. Of the 1099 VOC substrates that were present in at least 7 % of the subjects’ samples, a panel of 22 discriminated between healthy and CF subjects with 100 % specificity and 14 attributes correctly identified the 23 *P. aeruginosa*-positive compared to the 25 *P. aeruginosa*-negative subjects [22]. A proof of concept study evaluated a commercially available electronic nose device which operates on the principle of 32 polymer nanosensors measuring changes in electrical resistance by VOCs. The device provided discrimination between PCD and CF compared to healthy with equal sensitivity and specificity as differentiation between diseases—both around 84 % sensitivity and 60–65 % specificity. An indication of differences between stable and exacerbation state was noted but sample size was not designed to test this [23].

#### In vitro studies

Metabolomics has been applied to *P. aeruginosa* during *in vitro* growth to evaluate changes during chronic infection and to seek associations of bacterial metabolites with clinical outcomes. *P. aeruginosa* isolates collected longitudinally over several years from 18 patients were subjected to ^1^H NMR [24]. Growth characteristics and NMR profiles differed significantly between patients’ isolates and these differences exceeded differences associated with duration of infection. Conversely, analyses of all isolates by duration of infection showed nine metabolites that are associated with earlier vs. later stages of infection. Another study addressed metabolic adaptation of *P. aeruginosa* during chronic CF lung infection [25]. *P. aeruginosa* isolated from 13 adult CF patients were cultured in synthetic medium to imitate CF lung conditions, and culture supernatants were subjected to ^1^H NMR analyses. Principal component analyses (PCA) of the NMR spectra identified three dominant clusters. Analyses of these clusters relative to bacterial growth characteristics and patient outcomes showed significant associations between cluster membership and both, lung function and pH of the spent culture medium. Spent culture pH showed a negative correlation with lung function. These studies highlight that despite the limitations of imitating *in vivo* bacterial growth conditions, bacterial metabolomics can contribute to understanding the bacterial adaptation to the CF lung environment.

One study to date used bronchial epithelial cultures comparing pathways between CF and non-CF donors allowing insight into source of metabolites in respiratory secretions, i.e., epithelial cell derived, inflammatory cell derived, or of bacterial origin [26]. In a rigorous approach to reduce the effects of culture conditions, the investigators included cultures grown at three different study sites/methods. Differences in pathways were lower levels of several purines, reduced glucose metabolism in pentose and sorbitol pathways, and lower levels of oxidized and reduced glutathione. Kynurenine and anthranilate as metabolites of tryptophan pathways were increased in CF compared to non-CF.

#### Urine as matrix

Despite the ease of collection and description of urine metabolomic profiling in many other diseases, only one study has been reported in CF. This was in fact part of a targeted study on altered methyl status and oxidative stress in children with CF [27]. The urine NMR spectra showed significant elevation of phthalate compounds in pancreatic-insufficient children with CF but not in pancreatic-sufficient CF and non-CF children. The source of the phthalates was traced to the enteric coating of the enzymes. Although this study was designed to interrogate specific pathways, the untargeted metabolomic method led to changes in enzyme preparations as the authors reported in an addendum [28].

### Statistical approaches used in metabolomics

Two types of data analysis approaches, univariate analysis and multivariate analysis, have been widely used in metabolomics projects, including most of the CF related studies referenced in this paper.

Univariate analysis approach analyzes each metabolite separately. It includes parametric methods such as paired *t* test, Welch *t* test, and linear model and non-parametric methods such as Wilcoxon signed rank test, Mann-Whitney test, and Kruskal-Wallis ANOVA. A parametric method is used when the data basically meets normality assumption. Data transformations such as log transformation are often used to improve normality. *t* test is frequently used for the comparison of two classes, e.g., CF vs. non-CF. While *t* test is easy to use, one of the advantages of using a linear model is that multiple confounders can be controlled in the model, so that metabolic variations due to these confounders can be removed from the data. These linear models were for example used in the blood-based studies referenced here [6, 7]. Univariate analysis of metabolomics data usually includes hundreds of tests (one for each metabolite), therefore the control of false discovery rate (FDR) in multiple testing is very important. The most commonly used FDR control methods are *Q* value [29] and the Benjamini-Hochberg procedure [30].

Multivariate analysis approach analyzes all of the metabolites in the data simultaneously in one analysis. It detects important metabolic variations though dimension reduction. It includes non-supervised classification methods such as principal component analysis (PCA) and supervised classification methods such as partial least squares discriminant analysis (PLS-DA) and orthogonal partial least squares discriminant analysis (OPLS-DA). PCA detects major variations in the data without using sample classes, therefore being suitable for data exploration, and detection of outliers in the dataset. Most of the CF studies compare two or more classes of samples, e.g., CF vs. non-CF [6], CF with low inflammation vs. high inflammation [16], stable CF vs. unstable CF [19], and CF vs. PCD vs. healthy subjects [20], such studies benefit from using supervised classification methods. Unlike PCA which looks for major metabolic variations in the data, PLS-DA and OPLS-DA look for metabolic variations that are specifically related to the study classes. A major challenge of multivariate analysis is overfitting, i.e., the model fits the data so well that it cannot generalize to new data. Overfitting occurs when the model describes noise in the data. Statistical cross validation can be used to reduce overfitting. In cross validation, the samples are split into training set and testing set, and results from the training set are tested in the testing set as used in several of the studies here [6, 16, 19, 20, 25]. A limitation of cross validation is that all of the samples used for validation come from the same study. The most rigorous validation is external validation, i.e., validation performed in an independent data set. Montuschi et al. provided some good examples of using external validation to identify CF-related biomarkers [19, 20]. When an independent data set is not available, permutation-based validation could be used as an alternative, not used in any of the referenced CF studies. In permutation-based validation, the predictive power of the model is compared with the predictive powers calculated using hundreds and thousands of permutated data sets. Using it in combination with cross validation provides a more reliable validation result than using cross validation alone.

It is important to choose appropriate statistical method based on the purpose of the analysis and statistical assumptions. A summary of the statistical methods and their assumptions is in Table 1. The results generated from univariate analysis and multivariate analysis often complement each other. Examining results from both approaches allows researchers to look at the data from different perspectives and extract the best possible amount of information from the data.

**Table 1:** Common statistical approaches used in metabolomics data analysis

	Method	Purpose	Statistical assumptions^a^
A. Methods that analyze each metabolite separately			
Parametric methods	Paired *t* test	Compare two groups	Random sampling, normality, paired samples, no major outliers
	Student *t* test	Compare two groups	Random sampling, normality, independent samples, equal variances, no major outliers
	Welch *t* test	Compare two groups	Random sampling, normality, independent samples, unequal variances, no major outliers
	Linear model	Compare two or more groups and with the possibility to control confounders	Random sampling, linearity, and additivity, errors are independent, homoscedastic, and follow normal distribution, no major outliers
Nonparametric methods	Wilcoxon signed rank test	Compare two groups	Random sampling, paired samples, differences between paired samples have symmetrical distribution
	Mann-Whitney U test	Compare two groups	Random sampling, independent samples
	Kruskal-Wallis ANOVA	Compare more than two groups	Random sampling, independent samples
B. Methods that analyze all of the metabolites simultaneously			
Unsupervised classification methods	PCA	Detect major pattern in the data, detect outliers	Linearity
Supervised classification methods	PLS-DA	Find metabolites that best separate two or more study groups	Linearity, no major outliers
	OPLS-DA	Find metabolites that best separate two or more study groups, with easier result interpretation than PLS-DA	Linearity, no major outliers

^a^The assumption of continuous data is not listed, because all of the metabolomics data are continuous data and meet this assumption

## Conclusions

The ability of simultaneously obtaining an almost complete snapshot of metabolic activity in a given system is a strength of metabolomics, with the caveat that some metabolites may potentially require special processing methods, are not stable, or that markers of interest are proteins. Examples in CF include markers of oxidative stress or C-reactive protein. Several lessons have been learned to date; One, systemically measured metabolites, i.e., blood derived, confirm previously known pathways and add some potentially novel discoveries. Assessment of lung-specific markers may be challenging, especially during early disease when changes may be compartmentalized to the lung; two, samples directly obtained from the lungs as BAL or exhaled breath provide evidence for early inflammation but are still in early stages for use as biomarkers. Since targets in EBC are very dilute, contamination from oral or GI tract has to be monitored; three, detection of bacterial metabolites in human samples is complicated by substantial overlap between bacterial and host metabolites. Despite their obvious limitations, *in vitro* studies of bacteria may contribute to understanding of bacterial metabolism in CF lung infection. In summary, metabolomics remains a promising technology but study design, selection of participants, and validation of findings remain crucial [31].
